# Using equitable impact sensitive tool (EQUIST) to promote implementation of evidence informed policymaking to improve maternal and child health outcomes: a focus on six West African Countries

**DOI:** 10.1186/s12992-018-0422-1

**Published:** 2018-11-06

**Authors:** Chigozie Jesse Uneke, Issiaka Sombie, Henry Chukwuemeka Uro-Chukwu, Ermel Johnson

**Affiliations:** 1 African Institute for Health Policy and Health Systems, Ebonyi State University, CAS Campus, Abakaliki, PMB 053 Nigeria; 2 Organisation Ouest Africaine de la Santé, 175, Avenue Ouezzin Coulibaly, Bobo Dioulasso 01, 01 BP 153 Burkina Faso

**Keywords:** EQUIST, West Africa, Maternal, Neonatal, Child, Health, Equity

## Abstract

**Background:**

United Nations Children’s Fund (UNICEF) designed **EQU**itable **I**mpact **S**ensitive **T**ool (EQUIST) to enable global health community address the issue of equity in maternal, newborn and child health (MNCH) and minimize health disparities between the most marginalized population and the better-off. The purpose of this study was to use EQUIST to provide reliable evidence, based on demographic health surveys (DHS) on cost–effectiveness and equitable impact of interventions that can be implemented to improve MNCH outcomes in Benin, Burkina Faso, Ghana, Mali, Nigeria and Senegal.

**Methods:**

Using the latest available DHS data sets, we conducted EQUIST Situation Analysis of maternal and child health outcomes in the six countries by sub-national categorization, wealth and by residence. We then identified the poorest population class within each country with the highest maternal and child mortality and performed EQUIST Scenario Analysis of this population to identify intervention package, bottlenecks and strategies to address them, cost of the intervention and strategies as well as the number of deaths avertible.

**Results:**

Under-five mortality was highest in Atlantique (Benin), Sahel (Burkina Faso), Northern (Ghana), Sikasso (Mali), North-West (Nigeria), and Diourbel (Senegal). The number of under-five deaths was considerably higher among the poorest and rural population. Neonatal causes, malaria, pneumonia and diarrhoea were responsible for most of the under-five deaths. Ante-partum, intra-partum, and post-partum haemorrhages, and hypertensive disorder, were responsible for highest maternal deaths. The national average for improved water source was highest in Ghana (82%). Insecticide treated nets ownership percentage national average was highest in Benin (73%). Delivery by skilled professional is capable of averting the highest number of under-five and maternal deaths in the six countries. Redeployment/relocation of existing staff was the strategy with highest costs in Burkina Faso, Nigeria and Senegal. Ghana recorded the least cost per capita ($0.39) while the highest cost per capita was recorded in Benin ($4.0).

**Conclusion:**

EQUIST highlights the most vulnerable and deprived children and women needing urgent health interventions as a matter of priority. It will continue to serve as a tool for maximizing the number of lives saved; decreasing health disparities and improving overall cost effectiveness.

**Electronic supplementary material:**

The online version of this article (10.1186/s12992-018-0422-1) contains supplementary material, which is available to authorized users.

## Introduction

As the United Nations (UN) Millennium Development Goals (MDGs) initiative of 2000 rounded off in 2015, available reports indicated that most countries made various levels of progress towards achieving the MDGs 4 and 5 (reducing child mortality and improving maternal health, respectively) [1]. Recent statistics from the WHO Global Health Observatory showed that in Africa, infant, neonatal and under-five mortality rates per 1000 live births reduced from 93.5, 40.9 and 154 in 2000 to 53.9, 27.7 and 79.5 in 2015 respectively [2]. Similarly, the maternal mortality ratio (MMR) per 100,000 live births in Africa reduced from 840 in 2000 to 542 in 2015 [3]. Despite these appreciable, the current infant mortality rate (IMR), neonatal mortality rate (NMR), under-five mortality rate (U5MR) and MMR in Africa are still unacceptably high.

Of all the sub-regions of Africa, the West-Africa with a population of more than 357million (about 1/3 of entire African population) [4, 5], is among the worst performing regions in terms of addressing maternal and child mortality. The MMR of some of the West African countries are among the highest in the world such as Sierra Leone (1360/100,000), Nigeria (814/100,000), Liberia (725/100,000) and The Gambia (706/100,000) [5]. Similarly, the sub-region has countries which records U5MR that are among the highest in the world including Sierra Leone (118/1000), Mali (114/1000), Nigeria (108/1000) and Benin (100/1000) [5].

Recent studies sponsored by the West African Health Organization (WAHO) report that contextual barriers such as road conditions, culture, knowledge of risks and the status of women, and health systems barriers including geographic distance of health centres, services delivery organisation, the availability and ability of health services, and the quality of care, all act together to increase maternal and child mortality in the sub-region [6, 7]. It has been argued that any effort to improve maternal and child mortality in West Africa must not only focus on investing in interventions but more importantly on strengthening health systems and context to enable efficient and effective implementation of proven life-saving interventions [6]. Among the most critical health systems components that is often neglected in health systems strengthening efforts to improve MNCH outcomes in Africa is the concept of equity [8]. Wilunda and co-workers [9] have noted that dramatic inequities in maternal and child care in Africa are now being increasingly recognized and addressed in strategic documents, action plans and related indicators, but unfortunately are seldom translated into concrete actions.

Evidence abound which showed that decrease in maternal and child mortality in low and middle-income countries (LMICs) including the African region has been accompanied by increased inequity in health outcomes between the poor and those better off [10–13]. Consequently, the United Nations Children’s Fund (UNICEF) has strongly advocated against the ‘mainstream approach’ where scaling–up of health interventions favour wealthier groups in the society, but rather is promoting an ‘equity–focused’ approach in which interventions are targeted at the poorest in the society [14]. In a recent publication [13], UNICEF made a strong case for equitable investment and argued that since most maternal and child deaths in LMIC could have been prevented with practical, high-impact, and, low-cost health interventions, extending services to the most deprived and marginalized communities would not only avert more deaths, but would also do so more cost-effectively.

To this end, a number of tools have been developed to assess the relationship between cost–effectiveness and equitable impact in maternal and child mortality reduction [14]. Some of these tools included the Marginal Budgeting for Bottlenecks (MBB) [15], Choice of Interventions that are Cost–Effective (CHOICE) [16], and the Lives Saved Tool (LiST) [17]. According to Waters and colleagues [18], the major limitation of these tools is that they make no allowance for income-related inequalities in countries and therefore cannot fully address equitable impact considerations. To address this limitation, the UNICEF designed the **EQU**itable **I**mpact **S**ensitive **T**ool (EQUIST) to enable the global health community improve equity in MNCH and reduce health disparities between the most marginalized mothers and young children and the better-off [18, 19]. EQUIST is an online tool (http://equist.info/en/pages/home), which has been described as a medium-term strategic planning, modelling and monitoring platform that serves to improve child and maternal health as well as nutrition equity in LMICs [18–22].

The purpose of this study was to use EQUIST to provide reliable evidence, based on globally available demographic health surveys (DHS) on cost–effectiveness and equitable impact that will facilitate the implementation of interventions that will improve MNCH outcomes in West Africa with a focus on six West African countries (Benin, Burkina Faso, Ghana, Mali, Nigeria and Senegal). The goal was to provide decision makers and global health community with scientific information that will enable them think about issues of equity in MNCH in a more systematic and evidence-informed way, in order to design health intervention strategies that will lead to stronger, more resilient health systems in West Africa. In this study, we used EQUIST to: (i). create an accurate picture of the health status of the most deprived children and women in Benin, Burkina Faso, Ghana, Mali, Nigeria and Senegal; (ii). identify which populations are at greatest risk, why they are at risk, and how many lives can be saved with appropriate action; (iii). identify the highest impact, most cost-effective strategies to level disparities; and (iv). measure the potential effects in terms of lives saved and costs.

## Methods

### Setting

Geographically, the West African sub-region is bounded by the Atlantic Ocean in the west and by the Gulf of Guinea in the south and is characterised by a very rich ethnic and social diversity. Most of the countries of West Africa are classified as poor and their economies are not very well developed or diversified with the Human Development Index (HDI) rank among the poorest in the world [4]. The 2014 HDI rank of Benin, Burkina Faso, Ghana, Mali, Nigeria and Senegal out of 188 countries were 166, 183, 140, 179, 152, and 170 respectively [2]. The health situation in these six countries like all others in West Africa region is a reflection of the development stage which most of the countries in the region are at. These six countries were selected for this investigation because there are the countries in which the West African Health Organization is implementing the Moving Evidence to Policy (MEP) project in maternal and child health.

### Design

An explanation of the main concepts, assumptions and default data sources used in EQUIST are presented in the EQUIST technical note [23], while the step-wise procedure of performing the analysis is described in the EQUIST user’s guide [24]. EQUIST is linked to LiST, estimate cost using MBB, and uses data globally available such as DHS [24], and is based on a simple seven-step theory of change [21, 23]. This theory of change assumes that investments in, and implementation of, equity-focused strategies that remove quantifiable health system bottlenecks will lead to improvements in the coverage of high-impact health interventions and improved health outcomes for target populations [21].

### Analysis

#### EQUIST situational analysis

EQUIST is pre-loaded with DHS data sets and we used the latest available DHS data sets of the six West African countries we considered in this study. The DHS are country-wide household survey that are nationally-representative and which provide a wide range of systematic information on health indicators and health services. We used the 2011 DHS data set of Benin, 2010 of Burkina-Faso, 2014 of Ghana, 2013 of Mali, 2013 of Nigeria and 2014 of Senegal to perform both profile and frontier situational analysis [23]. We conducted a general EQUIST situation analysis of maternal and child health outcomes in the six countries by sub-national categorization, by wealth and by residence. We then identified the poorest population class within each country with the highest maternal and child mortality and performed EQUIST scenario analysis of this population in order to identify the intervention package, the bottlenecks and strategies to address them, the cost of the intervention and strategies as well as the number of deaths avertible and lives saved per US$ invested.

- Profile analysis

Using the EQUIST Profile analysis, we assessed the general extent, nature and implications of inequities as they affect MNCH in the six countries. Under the Demographic Parameters of the Sector Category, we examined under-five mortality and neonatal mortality with reference to the key drivers of inequity, the underlying factors that explain inequities (wealth quintile, geography, and location) and analysed the scale of inequity (deprivation mostly concentrated in poorest quintile and in rural areas). Under the Epidemiological Parameters of the Sector Category, we performed EQUIST Profile analysis to determine the key epidemiological causes and the specific number of under-five, neonatal and maternal mortality each of them causes.

Under the Theme Category, we also performed EQUIST Profile analysis of the percentage of Effective Coverage of maternal and child health interventions including: (i). Family care practices (WASH, ITNs/Environmental safety, neonatal/infant care); (ii). Preventive services (immunization plus); (iii). Curative services (IMNCI, delivery by skilled professionals, EMONC). We related these interventions and effective coverage to the six countries by wealth (poorest and richest) and by residence (rural and urban).

(b) Frontier analysis

Using the EQUIST Frontier analysis, we identified the factors most likely to drive inequity, and compared the number of Under-five and maternal deaths that could be averted in the poorest wealth quintile in the six West African countries. Under the Frontier, we performed two analyses.

First, we performed the Equity Frontier analysis to identify how many under-five and maternal lives that could have been saved if the six countries equalize coverage values for the least disadvantaged within the most disadvantaged population (poorest quintile). This was to enable us know the number of deaths that will be averted if the coverage gaps for the most disadvantaged population was equivalent to that of the richest in each of the countries’ context.

Second, we performed the Operational Frontier analysis to determine the number of under-five and maternal deaths that could be averted if effective coverage of evidence based high impact interventions are implemented and if their bottlenecks are reduced with the same proportion as observed in the most disadvantaged quintiles in best- performing countries.

#### EQUIST scenario analysis

We conducted EQUIST scenario analysis for the six selected West African countries by wealth focusing on the poorest quintile.

- Analysis of epidemiological priorities

Using the EQUIST epidemiological priorities we identified three categories of mortality and their main causes in the poorest quintile as follows: (i). Neonatal mortality (asphyxia, prematurity, sepsis, pneumonia, diarrhoea, tetanus); (ii). Post-neonatal and child mortality (diarrhoea, malaria, meningitis, pneumonia, asphyxia, sepsis, measles, tetanus, pertussis, prematurity); (iii). Maternal mortality (antepartum haemorrhage, complicated abortion, obstructed labour, postpartum haemorrhage, sepsis infection).

(b) Analysis of interventions

We identified the priority interventions with which to address the epidemiological issues we selected. The interventions are grouped in nine “packages” further grouped into three service delivery modes: family care practices, preventive services, and curative services.

(c) Analysis of bottlenecks, causes & recommendations

We identified the priority bottlenecks to implementing the interventions we selected. We related the priority bottle necks with the eight EQUIST scenario coverage determinants including: (i) Availability of commodities, (ii) availability of human resources, (iii) geographical accessibility, (iv) financial affordability, (v) sociocultural acceptability, (vi) initial utilization, (vii) adequate coverage, and, (viii) effective coverage. The bottleneck analysis framework in EQUIST assumes that eight conditions (coverage determinants) must be met to provide effective coverage of any health intervention [23]. Using the EQUIST bottleneck analysis, we determined the severity of bottlenecks based on the indicators used to measure the level of compliance with each condition for utilization, as well as the relationship between initial utilization, adequate coverage. For each intervention, we identified the coverage determinant, bottleneck, cause of the bottleneck and recommendations to address them.

(d) Analysis of enabling environment and strategies to address bottlenecks and their causes

We performed the analysis of the enabling environment which is classified into four (social norms; legislation/policy; budget/expenditure; management/coordination) and identified the direct causes. We also performed the analysis of the strategies classified into five health systems building blocks (financing; service delivery; medical products, vaccines and technologies; health workforce; governance/leadership; information) to address the bottlenecks.

(e) Analysis of impact and cost

The EQUIST impact and cost analysis was performed to determine the following: (i). The operational frontier for maternal, under-five and neonatal mortality: that is amenable deaths if the deprived population coverage value was equal to the best performing countries, (ii). The equity frontier for maternal, under-five and neonatal mortality: that is amenable deaths if the deprived population coverage value was equal to the non-deprived population coverage value; (iii). Amenable under-five and maternal deaths among the poorest by intervention package in the six West African countries; (iv). The cost of strategies to avert both maternal and under-five mortality; (v). The cost per capita averting the number of deaths.

## Result

### Outcome of situational analysis

The EQUIST profile analysis of under-five mortality by sub-national regions under the demographic parameters of the sector category in the six West African countries showed the regions recorded the highest mortality Atlantique (Benin), Sahel (Burkina Faso), Northern (Ghana), Sikasso (Mali), North West (Nigeria), and Diourbel (Senegal). The number of deaths/1000 live births in these regions were more than twice the number recorded in the regions with the lowest number of deaths/1000 live births and the values were considerably higher than the national average in the six countries (Table 1). The number of under-five deaths/1000 live births was considerably higher among the poorest compared to the richest and among the rural compared to the urban population (Table 1).

**Table 1:** Six West African Countries Under-five mortality by Sub-National Regions, Wealth and Residence

Country/Year	National Average Deaths/1000 live births	By Sub-National Region Deaths/1000 live births (Number of deaths)		By Resident Deaths/1000 live births (Number of deaths)		By Wealth Deaths/1000 live births (Number of deaths)	
		Highest	Lowest	Rural	Urban	Poorest	Richest
Benin/2014	109	Atlantique: 106(5495)	Littoral: 50(1263)	122(20,864)	94(15,196)	126(8342)	63(4636)
Burkina-Faso/2010	114	Sahel: 181(9573)	Centre-Est: 60(3121)	120(58,279)	80(11,748)	134(17,071)	74(7503)
Ghana/2014	64	Northern: 118(12,709)	Greater Accra: 50(5352)	80(32,677)	68(27,213)	98(17,667)	68(10,050)
Mali/2013	123	Sikasso: 156(16,253)	Bamako: 76(3086)	146(58,505)	83(17,856)	145(18,040)	79(8861)
Nigeria/2013	117	North West: 149(343,740)	South West: 72(26,126)	134(492,003)	80(241,110)	153(235,760)	59(82,647)
Senegal/2014	50	Diourbel: 86(5922)	Dakar: 38(3660)	71(25,950)	41(7938)	85(11,328)	26(2397)

The outcome of profile analysis of neonatal mortality by sub-national regions, wealth and residence under the demographic parameters of the sector category is summarized in Table 2. The number of neonatal deaths/1000 live births was consistently higher among the poorest compared to the richest and among the rural compared to the urban population except in Ghana. The six countries under-five mortality by epidemiological cause under the Sector Category is presented in Table 3. The four diseases responsible for most of the deaths are neonatal causes, malaria, pneumonia and diarrhoea. The rural dwellers as well as the poorest had higher under-five mortality numbers across the six West African countries (Table 3).

**Table 2:** Six West African Countries Neonatal mortality by Sub-National Regions, Wealth and Residence

Country/Year	National Average Deaths/1000 live births	By Sub-National Region Deaths/1000 live births (Number of deaths)		By Resident Deaths/1000 live births (Number of deaths)		By Wealth Deaths/1000 live births (Number of deaths)	
		Highest	Lowest	Rural	Urban	Poorest	Richest
Benin/2014	33	Atlantique: 29(1493)	Littoral: 18(439)	35(6011)	31(4953)	32(2332)	27(2008)
Burkina-Faso/2010	30	Est: 46(2920)	Centre-Nord: 21(1129)	31(15204)	26(3796)	29(3719)	22(2220)
Ghana/2014	29	Ashanti: 42(6646)	Upper East: 24(544)	29(11845)	33(13154)	32(5761)	40(5888)
Mali/2013	39	Sikasso: 51(5303)	Bamako: 32(1314)	44(17654)	31(6759)	45(5636)	35(3909)
Nigeria/2013	36	North West: 42(96145)	South West: 30(16085)	43(155964)	33(98646)	44(67185)	29(40872)
Senegal/2014	22	Diourbel: 42(2897)	Ziguinchor: 18(599)	28(10359)	17(3326)	28(3785)	12(1158)

**Table 3:** Six West African Countries Under-five mortality numbers by six major Epidemiological Causes

Country/ Year	Situational description	Neonatal causes	Injuries	Malaria	Meningitis	Pneumonia (U5)	Diarrhoea (U5)
Benin/2014	Atlantique	1493	140	1225	52	657	852
	Littoral	439	34	242	11	132	150
	Rural	6011	547	4409	184	2188	3327
	Urban	4954	375	3041	132	1676	2140
	Poorest	2332	237	2041	87	1072	1730
	Richest	2008	104	831	33	446	423
Burkina-Faso/2010	Sahel	1993	386	1997	352	2020	1480
	Centre-Est	958	177	689	73	346	369
	Rural	15,205	2848	12,623	1560	9109	8054
	Urban	3796	669	2574	349	1557	795
	Poorest	3720	787	3836	481	2935	2723
	Richest	2220	454	1449	258	1254	507
Ghana/2014	Northern	2576	682	3854	312	1923	1639
	Greater Accra	2669	170	968	60	670	340
	Rural	11,845	1622	6233	621	4718	3145
	Urban	13,154	1080	5206	415	2838	1842
	Poorest	5761	864	3815	344	2832	1898
	Richest	5888	255	1927	88	830	446
Mali/2013	Sikasso	5303	598	1635	235	1403	1254
	Bamako	1314	105	313	39	204	121
	Rural	17,652	1944	5351	1014	7071	6037
	Urban	6759	579	1620	298	1985	1041
	Poorest	5636	590	1674	382	2114	1697
	Richest	3910	286	792	157	774	249
Nigeria/2013	North-West	96,134	17,204	80,319	12,322	58,954	28,739
	South-West	13,775	973	4438	616	2295	1512
	Rural	155,975	49,628	101,344	15,527	76,233	57,657
	Urban	98,638	11,857	50,686	7174	29,437	14,586
	Poorest	61,186	10,668	51,099	7669	34,847	32,244
	Richest	40,867	3566	15,654	2058	8215	3361
Senegal/2014	Diourbel	2317	306	1094	84	843	457
	Dakar	1570	188	682	51	515	162
	Rural	10,359	1340	4566	370	3358	2354
	Urban	3327	416	1448	114	1113	416
	Poorest	3785	565	1993	171	1654	1576
	Richest	1158	113	420	29	321	67

The poorest and the rural dwellers across the six countries had the highest number of neonatal deaths (Table 4). The four diseases responsible for the highest neonatal mortality included sepsis, asphyxia, prematurity and congenital disorders. Prematurity was the major killer of the neonates in all the six countries with the mortality considerably higher among the rural compared to the urban population in Benin (2151 vs. 1622), Burkina Faso (4457 vs. 1175), Mali (4510 vs. 1828), Nigeria (49,314 vs. 33,432) and Senegal (2951 vs. 1125). An exception was in Ghana where the neonatal mortality due to prematurity was higher among the urban (4771) compared to the rural population (3680) (Table 4).

**Table 4:** Six West African Countries Neonatal mortality numbers by six major Epidemiological Causes

Country/ Year	Situational description	Congenital	Tetanus	Prematurity	Asphyxia	Pneumonia	Sepsis
Benin/2014	Atlantique	96	–	519	414	244	98
	Littoral	28	–	148	118	81	31
	Rural	396	–	2151	1746	859	357
	Urban	305	–	1622	1290	977	367
	Poorest	135	–	772	634	438	173
	Richest	130	–	671	524	384	140
Burkina-Faso/2010	Est	166	83	812	820	206	605
	Centre(exc.Ouag)	15	7	74	75	85	251
	Rural	909	457	4457	4501	953	2800
	Urban	239	120	1175	1186	203	627
	Poorest	218	109	1073	1084	245	706
	Richest	139	69	679	685	123	383
Ghana/2014	Ashanti	695	245	2329	2120	180	555
	Upper East	52	20	192	159	22	61
	Rural	1009	584	3680	3558	478	1826
	Urban	1299	446	4771	3914	474	1327
	Poorest	373	277	1503	1392	432	1516
	Richest	620	168	2273	1822	135	428
Mali/2013	Sikasso	249	176	1647	1566	304	874
	Kidal	7	8	53	53	2	5
	Rural	589	537	4510	3892	1519	5291
	Urban	311	184	1828	1735	490	1525
	Poorest	231	207	1722	1584	364	1069
	Richest	224	109	1220	1227	183	590
Nigeria/2013	North-West	4644	4714	32,048	29,044	4621	14,464
	South-West	927	256	4669	4957	493	1234
	Rural	7364	6338	49,314	45,540	9269	27,478
	Urban	5937	2219	33,432	33,222	4198	11,514
	Poorest	3117	3657	21,804	19,751	3534	10,771
	Richest	2914	619	14,065	15,276	1197	3013
Senegal/2014	Diourbel	327	94	890	861	145	431
	Kedougou	23	5	65	64	19	48
	Rural	1069	326	2951	2867	674	1981
	Urban	447	112	1125	1005	100	329
	Poorest	344	114	987	979	296	906
	Richest	158	35	398	347	32	113

The six diseases responsible for the highest maternal mortality included ante-partum haemorrhage, intra-partum haemorrhage, post-partum haemorrhage, hypertensive disorder, maternal sepsis and complicated abortion. Considerably higher maternal mortality was recorded in the rural compared to the urban population across all the countries except in Ghana (Table 5).

**Table 5:** Six West African Countries Maternal mortality numbers by six major Epidemiological Causes

Counntry/ Year	Situational description	Ante-partum	Intra-partum	Post-partum	Hypertensive	Maternal sepsis	Complicated abortion
Benin/2014	Atlantique	16	16	16	25	19	14
	Mono	6	6	6	9	7	5
	Rural	54	54	54	83	64	47
	Urban	51	50	50	78	60	44
	Poorest	23	23	23	36	27	20
	Richest	23	23	23	36	27	20
Burkina-Faso/2010	Boude du Mou.	23	3	42	45	29	27
	Centre(exc.Ouaga)	8	0.84	14	15	10	9
	Rural	170	18	308	324	209	195
	Urban	35	4	63	67	43	40
	Poorest	44	5	80	85	55	51
	Richest	36	4	65	68	44	41
Ghana/2014	Ashanti	46	46	46	71	55	40
	Upper East	7	7	7	10	8	6
	Rural	120	118	118	184	141	104
	Urban	117	116	116	180	137	101
	Poorest	53	52	52	81	62	46
	Richest	43	43	43	66	51	37
Mali/2013	Koulikoro	70	69	69	108	82	61
	Kidal	2	2	2	3	2	2
	Rural	223	220	220	342	262	193
	Urban	120	119	119	184	141	104
	Poorest	69	68	68	106	81	60
	Richest	96	62	62	96	73	54
Nigeria/2013	North-West	1726	1707	1707	2656	2030	1499
	South-West	273	270	270	420	321	237
	Rural	2737	2707	2707	4211	3218	2376
	Urban	2240	2215	2215	3446	2634	1945
	Poorest	1153	1140	1140	1774	1356	1001
	Richest	1052	1040	1040	1618	1237	913
Senegal/2014	Dakar	29	28	28	44	34	25
	Kedougou	2	2	2	3	2	2
	Rural	108	106	106	166	127	93
	Urban	58	57	57	89	68	50
	Poorest	39	39	39	61	46	34
	Richest	27	27	27	42	32	24

The percentage national average for WASH (improved water source) was higher in Ghana (82%), Mali (73%), and Senegal (81%), compared to the remaining three countries. ITN ownership percentage national average was highest in Benin (73%) and Mali (61%) but lowest in Nigeria (13%). The percentage of the ITN ownership was lower among the poorest compared to the national average in Benin (73% vs. 68%), Burkina-Faso (47% vs. 42%), Mali (61% vs. 56%), Nigeria (13% vs. 9%) and Senegal 43% vs. 38%) (Table 6). Ghana recorded the highest percentage of national average of exclusive breast feeding (52%) compared to the other five countries. The highest percentage coverage of DTP3 immunization was recorded in Burkina Faso (90%) and Ghana (89%), with the least in Benin (0.74%). In all the six countries, the percentage coverage of DTP3 immunization among the poorest was lower than the national average (Table 6). In terms of the curative services (essential care and case management of premature babies), the percentage coverage was low across the countries except in Benin (80% and 100% respectively), also the percentage coverage among the poorest was generally lower than the national average.

**Table 6:** Percentage of health intervention effective coverage by residence and wealth in Six West African Countries

Country/ Year	Situational description	Family Care Practices			Preventive Services	Curative Services		
		WASH (Improved water source)	ITNs (ITN ownership)	NIF (Excl breast feeding)	Immunization Plus (DTP3)	IMNCI (Oral antibiotic case mgt)	Delivery by skilled professionals (Essential care)	EMONC (Case Mgt of prematurity)
Benin/2014	National average	18	73	41	0.74	23	80	100
	Rural	7	72	42	0.72	26	78	100
	Urban	34	74	41	0.76	19	84	100
	Poorest	0	68	40	0.60	19	66	100
	Richest	58	77	39	0.88	13	91	100
Burkina-Faso/2010	National average	17	47	25	90	42	ND	25
	Rural	7	48	25	89	37	ND	25
	Urban	53	45	25	92	60	ND	25
	Poorest	100	42	25	83	29	ND	25
	Richest	59	48	25	93	52	ND	25
Ghana/2014	National average	82	47	52	89	43	16	NA
	Rural	74	55	52	89	39	20	ND
	Urban	89	36	52	88	51	6	ND
	Poorest	82	55	52	87	28	21	ND
	Richest	82	31	52	92	22	2	ND
Mali/2013	National average	73	61	36	63	37	26	13
	Rural	73	61	0.7	59	36	27	8
	Urban	73	58	1	79	40	25	39
	Poorest	73	56	41	48	22	25	2
	Richest	73	59	29	78	44	24	42
Nigeria/2013	National average	67	13	17	38	19	28	4
	Rural	54	13	17	25	16	20	1
	Urban	86	13	21	62	25	27	16
	Poorest	36	9	17	7	14	6	0
	Richest	84	12	17	80	35	27	29
Senegal/2014	National average	81	43	32	89	33	27	7
	Rural	72	44	33	89	40	28	3
	Urban	91	42	32	89	28	27	4
	Poorest	56	38	28	86	34	27	2
	Richest	97	33	23	92	40	26	9

The outcomes of the analysis of avertible deaths by epidemiological cause and equity/operational frontier for under-five children in the six countries are shown in Table 7. In the Burkina Faso and Nigeria, the three main diseases responsible for the highest number of avertible under-five deaths by equity and operational frontiers are malaria, pneumonia, and diarrhoea. In Ghana, Mali and Nigeria, the four main diseases responsible for the highest number of avertible maternal deaths by equity and operational frontiers are sepsis, hypertensive disorders, post-partum haemorrhage and intra-partum haemorrhage (Table 7).

**Table 7:** Avertible deaths among the poorest by epidemiological cause and equity/operational frontier for under-five mortality in six West African countries

Main causes of deaths avertible	Benin		Burkina-Faso		Ghana		Mali		Nigeria		Senegal	
	Equity	Operational	Equity	Operational	Equity	Operational	Equity	Operational	Equity	Operational	Equity	Operational
Under-five deaths												
Malaria	174	853	1313	1745	392	1352	42	940	4325	31,406	0	1240
Measles	0	0	216	169	12	3	169	99	1342	805	58	16
Pneumonia (U5MR)	88	299	1026	862	204	792	609	802	11,103	15,381	250	450
Diarrhoea (U5MR)	772	1100	2053	2016	545	911	1285	979	24,401	19,849	1231	998
Tetanus	0	0	11	21	194	−3.46	101	34	2926	1169	37	10
Prematurity	126	259	4	639	396	874	559	1009	5798	13,161	509	521
Asphyxia	489	136	5	635	1308	653	876	938	8693	12,059	217	536
Sepsis	38	94	144	563	770	1251	511	868	4721	8385	547	687
Maternal deaths												
Complicated abortion	8	0.89	0	27	0	16	32	34	154	0	10	16
Sepsis	17	0	0	27	49	23	52	37	681	657	14	19
Hypertensive disorders	26	5	0	47	73	42	71	57	1271	926	16	31
Post-partum haemorrhage	17	0	2	46	54	21	44	39	636	656	13	22
Intra-partum haemorrhage	12	3	0.1	2	44	22	33	31	457	527	2	20
Ante-partum haemorrhage	12	3	0.94	20	44	22	33	32	462	533	2	20

### Outcome of scenario analysis

The number of amenable/avertible under-five deaths if the deprived population coverage value was equal to (i). the best performing countries (operational frontier) and (ii) the non-deprived population coverage value (equity frontier) are shown in Table 8. An additional chart file of the six West African countries shows this in more detail [see Additional files 1, 2, 3, 4, 5, 6]. In the six countries, pneumonia, diarrhoea and asphyxia were responsible for the highest number of amenable under-five deaths by operational and equity frontiers.

**Table 8:** Amenable deaths among the poorest by epidemiological cause and equity/operational frontier in six West African countries

Main causes of deaths amenable	Benin		Burkina-Faso		Ghana		Mali		Nigeria		Senegal	
	Equity	Operational	Equity	Operational	Equity	Operational	Equity	Operational	Equity	Operational	Equity	Operational
Under-five deaths												
Pneumonia (U5MR)	7	8	600	613	9	18	399	602	5644	9082	9	4
Diarrhoea (U5MR)	222	277	842	638	195	373	468	181	3311	–	245	101
Asphyxia	479	471	155	604	1140	638	739	803	8163	10,104	200	393
Malaria	172	243	193	440	–	–	35	345	745	14,599	–	–
Sepsis	19	19	–	–	240	90	175	117	1832	1287	–	–
Prematurity	55	55	58	60	91	67	111	109	1796	1893	54	34
Measles	–	–	135	67	9	–	105	31	910	246	32	46
Tetanus	–	–	–	–	63	22	50	30	900	549	–	–
Pertussis	–	–	–	–	27	–	42	28	1827	593	38	40
Neonatal deaths												
Asphyxia	479	471	155	604	1140	638	739	803	8163	10,104	200	393
Sepsis	19	19	2	4	240	90	275	117	1832	1287	–	–
Prematurity	55	55	58	60	91	67	111	109	1796	1893	54	34
Pneumonia (NNMR)	2	2	–	1	–	–	–	4	–	–	–	–
Diarrhoea (NNMR)	6	7	28	21	8	16	23	9	–	–	–	–
Tetanus	–	–	–	–	63	22	50	30	900	549	–	–
Maternal deaths												
Ante-partum haemorrhage	12	12	–	21	39	20	24	30	309	508	2	14
Intra-partum haemorrhage	12	12	–	2	39	19	24	30	306	503	2	14
Post-partum haemorrhage	15	12	0.75	38	31	19	21	30	283	500	2	14
Hypertensive disorders	9	8	–	27	48	20	29	29	353	490	3	15
Sepsis	–	–	–	–	26	10	33	19	533	317	–	–

Asphyxia is responsible for the highest number of amenable neonatal deaths by operational and equity frontiers in the six countries. Ante-partum haemorrhage, intra-partum haemorrhage, post-partum haemorrhage and hypertensive disorders are the diseases responsible for the highest number of amenable maternal deaths among the poorest quintile in Ghana, Mali and Nigeria (Table 8).

Amenable deaths among the poorest by intervention package and equity/operational frontier for under-five and maternal mortality in six West African countries are shown in Table 9. Delivery by skilled professional is a major intervention capable of averting the highest number of under-five and maternal mortality in all the six countries. IMNCI, ITNs/Environmental safety, WASH and Immunization plus are capable of averting under-five deaths ranging from 35 in Ghana to 15,599 in Nigeria. An additional chart file of the six West African countries shows this in more detail [see Additional files 1, 2, 3, 4, 5, 6].

**Table 9:** Amenable deaths among the poorest by intervention package and equity/operational frontier for under-five and maternal mortality in six West African countries

Main intervention package	Amenable under-five deaths by package and equity/operational frontier among the poorest											
	Benin		Burkina-Faso		Ghana		Mali		Nigeria		Senegal	
	Equity	Operational	Equity	Operational	Equity	Operational	Equity	Operational	Equity	Operational	Equity	Operational
Delivery by skilled professional	548	537	208	653	1534	817	1070	1014	12,654	13,116	254	426
IMNCI	–	–	554	576	–	–	375	588	5500	8985	–	–
ITNs/Environmental safety	182	258	202	462	–	–	42	409	796	15,599	–	–
WASH	236	293	941	711	216	412	544	206	3611	–	265	109
Immunization plus	–	–	161	66	35	–	141	56	2727	835	67	86
Amenable maternal deaths by package and equity/operational frontier among the poorest-												
Delivery by skilled professional	55	56	–	96	202	98	140	152	1892	2547	10	61

The cost of intervention strategies to avert the mortality as provided by the EQUIST impact and cost analysis are presented in Table 10. The strategies with the highest costs in Burkina Faso, Nigeria and Senegal are Redeployment/relocation of existing staff. Ghana recorded the least cost per capita ($0.39) while the highest cost per capita was recorded in Benin ($4.0) (Table 10). The avertible under-five, neonatal and maternal mortality by cause and by intervention package as well as the estimates of cost generation for the analysis and cost per capta of avertible number of deaths in the scenario in all the six countries are shown in more detail as an additional chart file [see Additional files 1, 2, 3, 4, 5, 6].

**Table 10:** Cost of intervention in USD ($) to avert mortality among the poorest by in six West African countries

Intervention strategy	Cost of intervention in six countries					
	Benin	Burkina Faso	Ghana	Mali	Nigeria	Senegal
Conditional cash transfer	521,950	1,173,987	424,339	1,205,482	9,210,783	987,425
Vouchers	521,950	1,173,987	424,339	1,205,482	9,210,783	987,425
Health insurance	521,950	1,173,987	424,339	1,205,482	9,210,783	987,425
Supply-side financial incentives	1,596,353	499,389	225,433	745,928	1,741,842	21,681
Pharmaceutical cost control	–	–	34,526	–	–	–
Community education & outreach	218,692	131,753	119,012	1460	3,977,842	230,580
Redeployment/relocation of existing staff	–	2,327,896	138,773	–	22,056,700	2,209,502
Leadership and management training	3,104,019	971,035	93,930	–	–	42,158
Health systems accountability	3,104,019	971,035	93,930	–	3,386,914	42,158
Task-shifting/task sharing	–	1,163,948	69,386	–	11,028,350	1,104,751
Ensure timely procurement of key commodities	–	–	13,810	76,891	40,574	3027
Pharmaceutical stock management	–	–	13,810	76,891	–	–
Pre-service training/recruitment	–	11,272	–	–	–	–
Pharmaceutical quality regulation	–	–	–	48,454	–	–
Cost per capita	4.0	3.0	0.39	2.0	2.0	2.0

## Discussion

The outcome of the EQUIST analysis generally showed that the six West African countries have unacceptably high maternal and child mortality that is perhaps among the worst in the world. Santi and Weigert [25] noted in their report that West Africa lags behind the other regions of Africa in terms of women health as gender-disaggregated indicators showed that the status of women has hardly improved. The regions that consistently recorded the highest maternal, under-five and neonatal mortality included Atlantique in Benin, Sikasso in Mali, North West in Nigeria, and Diourbel in Senegal. Using the EQUIST analysis, the picture of the health status of these regions were highlighted as having the most vulnerable and deprived children and women and needing urgent health interventions as a matter of priority.

Available reports from Nigeria support the outcome of the EQUIST situational analysis which indicated the highest maternal and child mortality in the North-West region of the country [26]. The Nigeria National Population Commission (NPC), noted in a previous report that there exist substantial variations across the Nigeria’s six geopolitical zones in terms of social, cultural, and economic status [27]. The North-East and North-West regions of Nigeria are noted to be characterized by high level of non-formal education, polygamous marriage, early/teenage marriage/pregnancy, poor access and utilization of modern health facility, and very high proportion of extremely poor rural population [28]. These factors contribute significantly to the very poor MNCH outcomes in the North-West and North-East Nigeria [26–28].

In Mali, a recent report confirms that the *Sikasso* is among the regions with the worst health statistics*, having* the highest *infant mortality* rate and *under-five mortality* rate in the country [29]. According to Daou and colleagues, the main factors that are significantly associated with child mortality in Sikasso Mali, included the level of education of parents, the age of the mothers and the lack of skilled health care resources [30]. In Senegal, and especially in Diourbel region, child marriage is reported to be a major contributor to the very high maternal and child mortality [31, 32].

The EQUIST situational analysis showed that the rural dwellers and poorest quintile population had consistently higher maternal and child mortality in all the six countries. Also, higher number of deaths due to epidemiological causes were recorded among the rural dwellers and poorest quintile population. In an earlier report on maternal mortality and access to obstetric services in West Africa, Ronsmans and co-workers noted that most rural women give birth at home in the absence of skilled care, while urban women tend to give birth in a hospital with a skilled attendant, consequently, maternal mortality is extremely high in rural areas, and substantially lower in urban areas [33]. In addition to this, higher maternal and child deaths are recorded in West African rural areas because of low quality of services at government facilities, inadequate outreach services, self-medication and client preferences for traditional medicine because traditional beliefs and practices remain strong in the region [34].

The EQUIST situational analysis showed that neonatal causes, malaria, pneumonia, and diarrhoea were the major causes of under-five mortality in all the six countries. Result also showed that sepsis, asphyxia, and prematurity were the leading causes of neonatal mortality in the countries. Both under-five mortality and neonatal mortality were highest among the poorest and the rural dwellers in the six countries. Available evidence from previous studies conducted in Nigeria indicated that these diseases were responsible for the highest under-five and neonatal mortality numbers in various parts of the country especially among the rural and the poorest [26, 27]. A similar trend was also reported in other West African countries [35].

EQUIST analysis showed that obstetric hemorrhage is the leading cause of maternal mortality in all the six countries. Of the three types of Obstetric haemorrhage, post-partum haemorrhage was responsible for the highest number of maternal mortality. In a previous study on maternal mortality undertaken in northern Nigeria, post-partum haemorrhage was a leading cause of maternal mortality, contributing 75% of the cases [36]. Reports from Mali, Senegal and other parts of West Africa, noted that post-partum haemorrhage was a major cause of maternal death [37, 38].

The EQUIST situational and scenario equity and operational frontier analysis showed that thousands of under-five and maternal deaths could have been averted if the countries had equalized coverage values for the least disadvantaged within the most disadvantaged population (poorest quintile). According to the UN Chronicle report, universal coverage of scientifically proven cost-effective interventions are capable of reducing child deaths from about 2 million to just 650,000 [39]. According to Partnership for Maternal, Newborn & Child Health, the majority of maternal and child mortality and morbidity are preventable with interventions that are effective and affordable which prevent or treat the most common causes of illness [40]. A recent World Bank report indicated that scaling up all interventions in the packages of maternal and newborn health, plus folic acid before pregnancy, and child health from the existing rate of coverage to 90% would avert 149,000 maternal deaths; 849,000 stillbirths; 1,498,000 neonatal deaths; and 1,515,000 child deaths [41]. There is therefore, an international consensus that improving the coverage and quality of these interventions should be the focus of policies and associated programmes [40].

In terms of the coverage of intervention package to improve maternal and child health outcomes, EQUIST analysis revealed that Ghana, Senegal and Mali had higher national average of 82%, 81% and 73% respectively compared to the remining three countries. A recent report indicated that Ghana achieved its Millennium Development Goal (MDG) water target about a decade before the 2015 deadline [42]. As in Ghana, substantial donor support as well as institution of effective water policy reforms are major contributors to the very high percentage national water coverage also witnessed in Senegal and Mali [43, 44].

The EQUIST analysis indicated that the percentage of the ITN ownership, DTP3 immunization and curative services such as essential care and case management of premature babies was lower among the poorest compared to the national average in most of the countries. A recent study on equity trends in ownership of ITNs involving eight West African countries (including Benin, Burkina Faso, Mali, Nigeria, Senegal etc.) and 11 other sub-Saharan African countries, showed that richer households were more likely to own ITNs than the poorest households [45]. The relatively lower percentage of ITNs possession by the population of the poorest quintile has been attributed to their inability to afford the cost of the ITNs and probably as a result of low access to health care among the poorest populations [46]. Similarly, regarding routine immunization, disparities in coverage within countries also exist between poor and wealthy populations, where children in poor households are at much greater risk of dying from vaccine-preventable diseases than children in relatively wealthier households [46, 47].

The EQUIST scenario analysis for mortality by intervention package showed that delivery by skilled professionals has the potential of averting the highest number of under-five and maternal mortality in all the six countries. Findings from a number of previous studies have shown that between 13 and 33% of maternal deaths and up to 25% of newborn deaths could be averted by the availability of skilled attendant at delivery [48, 49]. According to UNICEF, the very high NMR, IMR and U5MR recorded in LMICs are caused by diseases and medical conditions which can easily be prevented by skilled care during delivery and immediate neonatal period [50].

One of the major strengths of the EQUIST scenario analysis is the estimation of the actual cost of the strategies to avert the mortality. Redeployment/relocation of existing staff and task-shifting/task sharing are the strategies with the highest costs in Burkina Faso, Nigeria and Senegal. According to Santi and Weigert, the health sector is skilled-labour-intensive and in all countries of the West African region, the increase and effective management of health staff is crucial to the improvement of health systems [25]. Insight from the EQUIST analysis can help to prioritize this domain, of which emphasis must be laid on territorial equity in order to address the human resource shortage in rural areas, where the poorest people live but which still harbour the greatest health risks [25]. EQUIST scenario analysis also showed the cost per capita averting the number of deaths was least in Ghana ($0.39) and highest in Benin ($4.0). This outcome was not a surprise, among the six countries in this study, Ghana had the least total expenditure on health as percentage GDP, least private expenditure on health as percentage of total expenditure on health and least poverty headcount ratio and the best Human Development Index rank [5].

Carrera and colleagues [12] have noted that a better understanding of the effectiveness, impact, and costs of the operational strategies and service delivery modes that can be used to overcome existing bottlenecks (especially those faced by people living in low-income and lower-middle-income countries) is required to ensure that deprived populations receive low cost, high impact interventions.

### Study limitation

The major limitation of this study is that it is entirely based on DHS data. The weakness of information collected in DHS are well-established [51, 52]. One important weakness is that virtually all data obtained through DHS is subject to reporting and recall biases. Furthermore, DHS can only measure limited health indicators and health services and so misclassification biases are known to occur and the magnitude of the bias is often unknown and very difficult to correct [51]. As can be seen from this study, some of the DHS information from some of the countries could not be obtained for others due to the challenges associate with data collection process from the various target populations. Despite the limitation, DHS provide valuable and high-quality data base on various of health indicators in LMICs which in many cases are the only reliable source of scientific information for health policymaking and implementation.

## Conclusion

Although EQUIST can be described as a valuable tool which can assist decision makers to engage equity-focused approaches to improving MNCH outcomes, the knowledge and application of the tool is not yet wide-spread in LMICs. This study is the first attempt to demonstrate the usefulness of the EQUIST in the provision of scientific evidence on equity–focused approaches to health interventions to improve MNCH outcomes in West Africa. It has been argued that this type of information is very crucial as it supports an evidence-based prioritization of vulnerable populations and priority interventions, as well as an initial understanding of the broad health system issues that will need to be addressed in order to reduce health disparities in a region like West Africa [23].

Throughout the world and especially in LMICs, policy makers and other key stakeholders in the health sector have come to the realization that resources for scaling up cost–effective MNCH interventions in their populations are scarce. Consequently, they are faced with the complex task of identifying and implementing the most efficient and cost-effective interventions that will results to more deaths averted per fixed investment [53, 54]. According to Sridhar and co-workers [51], health investors usually like to know how many deaths (or episodes of disease) could be averted for a fixed level of investment. EQUIST not only provides this vital information but also disaggregates data to reveal inequities that are often masked by national averages [13].

EQUIST will continue to serve as a tool for maximizing the number of lives saved; decreasing health disparities and improving overall cost effectiveness [23]. The importance of this cannot be overstated because even if current rates of decline in under-five mortality are sustained, without additional investment in reaching the poorest, nearly 70 million newborns, infants and young children will still die from preventable causes by 2030 [13]. In this study EQUIST analysis has helped in identifying the country’s populations that are disadvantaged, why they are disadvantaged, and which combination of evidence based high impact interventions and health system strengthening strategies would produce the best results. The West African policymakers will find this very valuable. We recommend EQUIST to national decision makers in LMICs who are interested in conducting an in-depth analysis of the situation of the disadvantaged or underserved populations in their countries. If the national decision makers are also interested in bridging implementation gaps and in the development of policies that are based on a thorough assessment of how the health system is functioning, particularly with regards to producing equitable health outcomes, then EQUIST is highly imperative.

## Additional files

Additional file 1: Outcome of EQUSIT Scenario analysis for poorest quintile in Benin. (PDF 345 kb)

Additional file 2: Outcome of EQUSIT Scenario analysis for poorest quintile in Burkina Faso. (PDF 345 kb)

Additional file 3: Outcome of EQUSIT Scenario analysis for poorest quintile in Ghana. (PDF 345 kb)

Additional file 4: Outcome of EQUSIT Scenario analysis for poorest quintile in Mali. (PDF 345 kb)

Additional file 5: Outcome of EQUSIT Scenario analysis for poorest quintile in Nigeria. (PDF 345 kb)

Additional file 6: Outcome of EQUSIT Scenario analysis for poorest quintile in Senegal. (PDF 345 kb)
