# Supplementary material for: Using equitable impact sensitive tool (EQUIST) to promote implementation of evidence informed policymaking to improve maternal and child health outcomes: a focus on six West African Countries
Source: Global Health. 2018 Nov 6;14:104. doi: 10.1186/s12992-018-0422-1 (PMC6219200; doi:10.1186/s12992-018-0422-1)
Supplement: Supplementary file 1 — Outcome of EQUSIT Scenario analysis for poorest quintile in Benin. (PDF 345 kb) [file 12992_2018_422_MOESM1_ESM.pdf]

Additional File 1. Outcome of EQUSIT Scenario analysis for poorest quintile in Benin

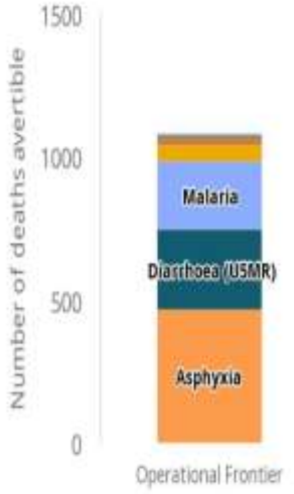

Fig 1a. Avertible under-fve mortality by cause

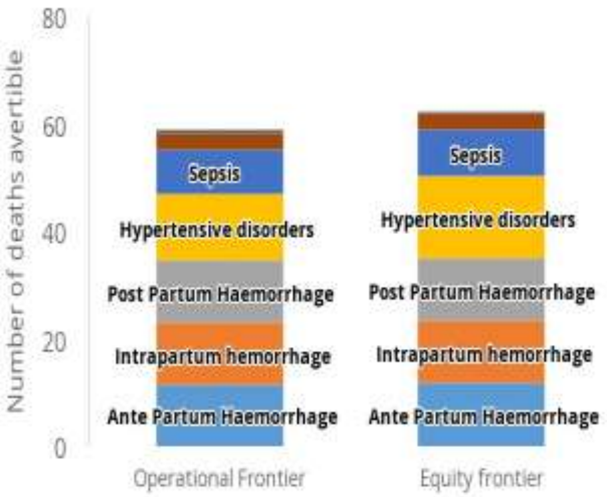

Fig 1b. Avertible maternal mortality by cause

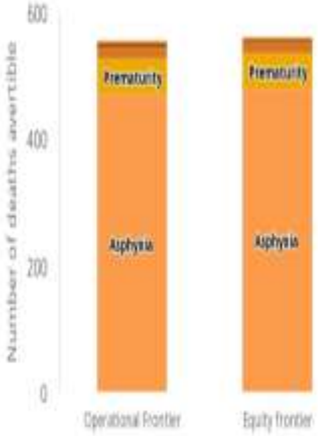

Fig 1c. Avertible neonatal mortality by cause

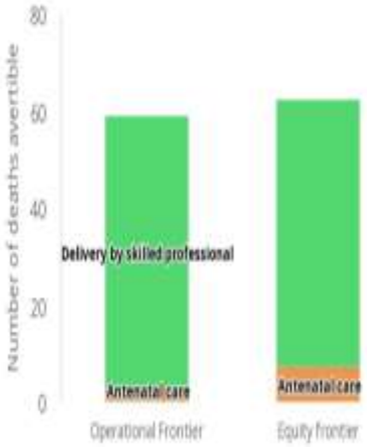

Fig 1d. Avertible maternal mortality by intervention package

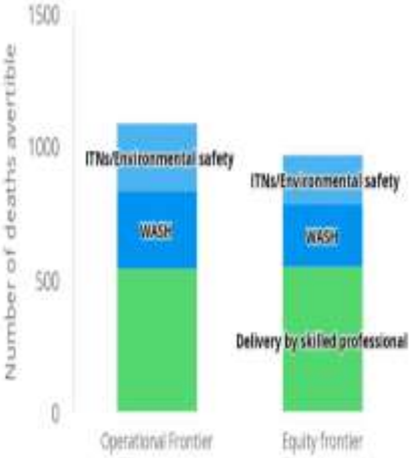

Fig 1e. Avertible under-five mortality by intervention package

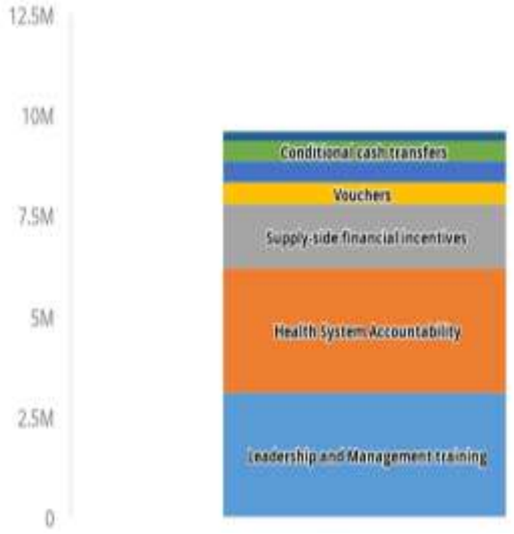

Fig 1f. Estimates of cost generation for the analysis

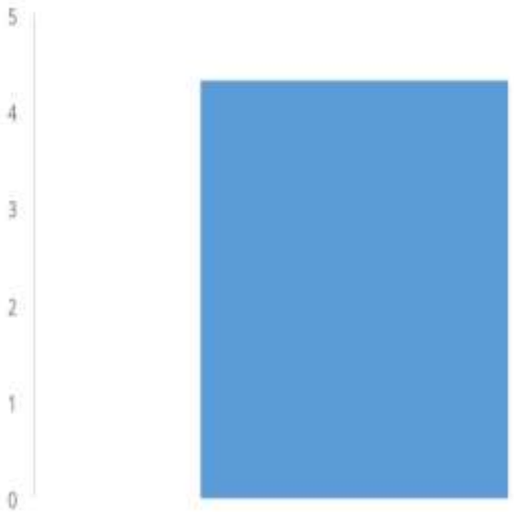

Fig 1g. Cost per capta of avertible number of deaths in the scenario
